# AMPK-Orchestrated Metabolic Reprogramming in Some Flavivirus Infections: Mechanisms and Therapeutic Opportunities

**DOI:** 10.3390/v18080910

**Published:** 2026-08-19

**Authors:** Kaci Craft, Imaan Muhammad, Shaokai Pei, Qiyi Tang

**Affiliations:** Department of Microbiology, Howard University College of Medicine, Washington, DC 20059, USA; kaci.craft@bison.howard.edu (K.C.); imaan.muhammad@bison.howard.edu (I.M.); shaokai.pei1@bison.howard.edu (S.P.)

**Keywords:** AMPK, flavivirus, Zika virus, dengue virus, metabolic reprogramming, lipid metabolism, lipid droplets, autophagy, mitochondrial dynamics, innate immunity, host-targeted antiviral therapy

## Abstract

5′-Adenosine monophosphate-activated protein kinase (AMPK) is the principal cellular energy sensor that coordinates metabolic adaptation by balancing anabolic and catabolic pathways in response to energic stress. Beyond its canonical role in maintaining energy homeostasis, AMPK has emerged as a central regulator of host–pathogen interactions by integrating lipid metabolism, autophagy, mitochondrial dynamics, oxidative stress, and innate immune signaling. Flaviviruses, including dengue virus, Zika virus, West Nile virus, Japanese encephalitis virus, and yellow fever virus, extensively remodel host metabolism to establish productive infection. As a master regulator of cellular metabolism, AMPK can either restrict or facilitate flavivirus replication in a context-dependent manner by regulating lipid droplet biogenesis, fatty acid synthesis and beta-oxidation, autophagy, mitochondrial homeostasis, and interferon-mediated antiviral responses. Conversely, flaviviruses actively manipulate AMPK signaling and its downstream metabolic networks to promote endoplasmic reticulum remodeling, replication organelle biogenesis, energy production, and immune evasion. In this review, we summarize recent advances in understanding the multifaceted roles of AMPK during flavivirus infection, with an emphasis on its regulation of metabolic reprogramming, organelle remodeling, and antiviral immunity. We further discuss the therapeutic potential of pharmacologically targeting AMPK and its downstream pathways as a host-directed strategy for broad-spectrum antiviral intervention against flaviviruses.

## 1. Introduction

Flaviviruses comprise a group of positive-sense single-stranded RNA (+ssRNA) viruses that include several medically important human pathogens, such as dengue virus (DENV), Zika virus (ZIKV), West Nile virus (WNV), Japanese encephalitis virus (JEV), and yellow fever virus (YFV). Together, these viruses cause millions of infections annually and remain major global public health threats owing to their widespread mosquito-borne transmission, substantial morbidity and mortality, and the limited availability of effective antiviral therapies. Like other RNA viruses, flaviviruses depend extensively on host cellular machinery to complete their replication and assembly. Following infection, they induce profound metabolic reprogramming, redirecting host nutrients and energy fluxes to favor viral replication complex (RC) formation, viral RNA synthesis, and virion production [1].

5′-Adenosine monophosphate-activated protein kinase (AMPK) is a heterotrimeric serine/threonine kinase that functions as the master regulator of cellular energy balance [2]. AMPK is activated during conditions of energy stress, including nutrient deprivation, hypoxia, oxidative stress, and mitochondrial dysfunction, conditions that increase the intracellular AMP/ATP or ADP/ATP ratio. Once activated, AMPK phosphorylates numerous downstream substrates to inhibit ATP-consuming anabolic processes, including fatty acid, cholesterol, and protein synthesis, while promoting ATP-generating catabolic pathways such as fatty acid beta-oxidation, autophagy, mitochondrial biogenesis, and glucose uptake [3]. Through these activities, AMPK integrates cellular metabolism with redox homeostasis, organelle quality control, and innate immune signaling, enabling cells to adapt to changing metabolic demands.

Emerging evidence indicates that AMPK is a pivotal regulator of virus–host interactions. During flavivirus infection, AMPK sits at the intersection of several cellular pathways that are extensively remodeled by the virus, including lipid metabolism, autophagy, mitochondrial function, and antiviral immunity. Flaviviruses manipulate these pathways to generate replication organelles, expand intracellular membrane networks, optimize energy production, and evade host immune defenses. For instance, DENV and ZIKV reprogram lipid metabolism to promote the formation of ER-derived replication complexes through coordinated regulation of acetyl-CoA carboxylase (ACC), sterol regulatory element-binding protein (SREBP), and lipid droplet dynamics [1,4]. Conversely, activation of AMPK can suppress viral replication by inhibiting lipogenesis, promoting fatty acid oxidation and autophagy, preserving mitochondrial integrity, and enhancing interferon signaling and interferon-stimulated gene (ISG) expression [5]. However, the outcome of AMPK activation is highly context-dependent, varying with the infecting virus, cell type, stage of infection, and metabolic status of the host cell.

In this review, we discuss how AMPK orchestrates metabolic reprogramming during flavivirus infection and examine its emerging role as a central metabolic hub linking lipid metabolism, organelle homeostasis, and innate immunity. We first summarize the molecular architecture, activation mechanisms, and signaling functions of AMPK. We then examine how flaviviruses reprogram AMPK-dependent pathways to remodel lipid metabolism, autophagy, mitochondrial dynamics, and antiviral immune responses. Finally, we evaluate current progress in the development of AMPK-targeted therapeutics and discuss the opportunities and challenges of exploiting host metabolic pathways as broad-spectrum antiviral strategies against flavivirus infections.

## 2. Overview of AMPK Signaling and Function

### 2.1. Structure and Functional Organization of AMPK

**Figure 1:**
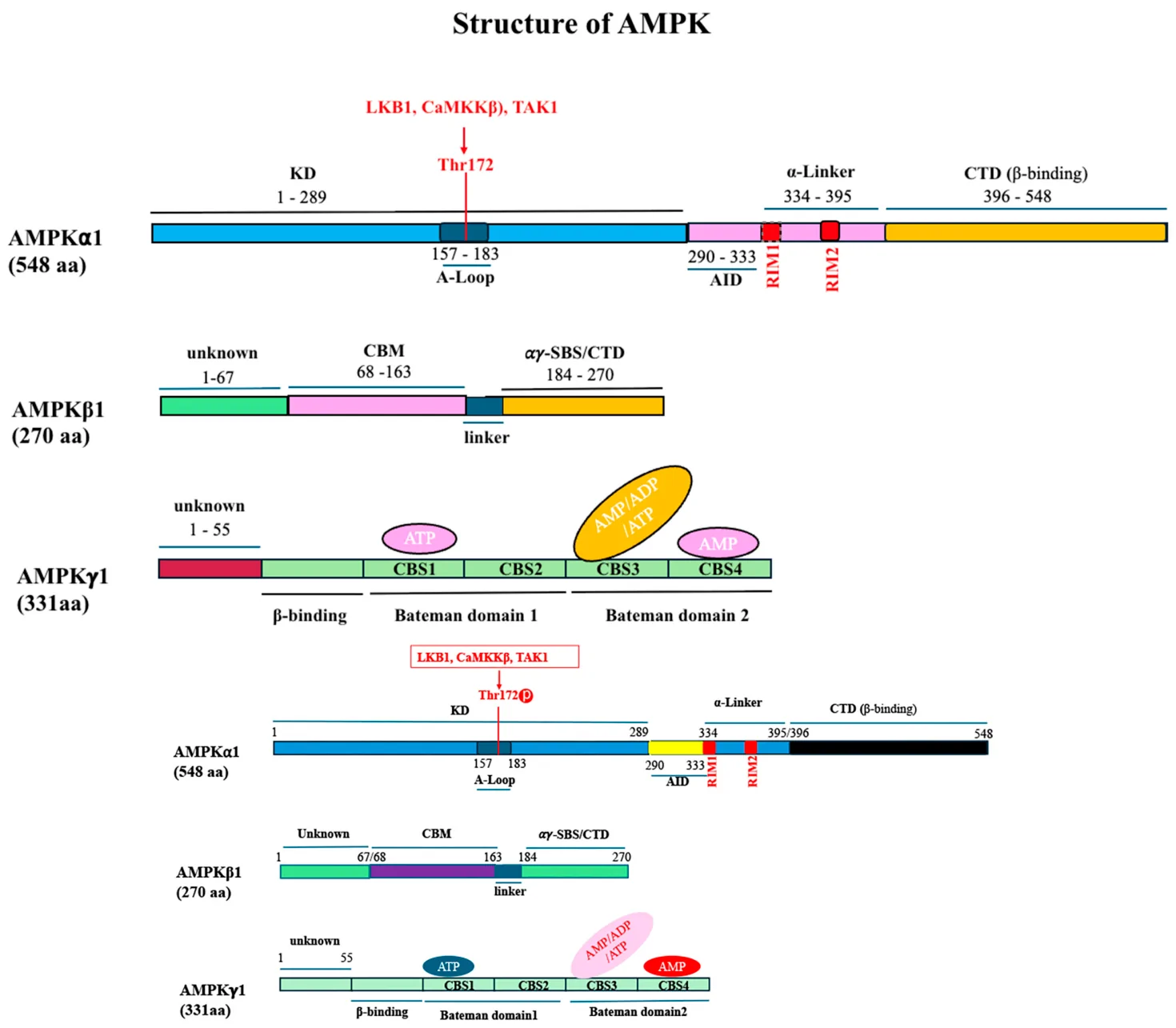
Functional domains of AMPK proteins. Shown is a representative AMPK α1β1γ1 heterotrimer. Although AMPK exists as 12 distinct heterotrimeric complexes generated by different α, β, and γ isoform combinations, the overall domain architecture is highly conserved across isoforms; the residue numbering shown corresponds specifically to the α1, β1, and γ1 proteins. The α subunit (AMPKα1, 548 aa) contains a kinase domain (KD; residues 1–289) with an activation loop (A-loop; residues 157–183) phosphorylated at Thr172 by LKB1, CaMKKβ, and TAK1, followed by an autoinhibitory domain (AID; residues 290–333), two regulatory subunit-interacting motifs (RIM1 and RIM2), an α-linker that mediates communication between the γ subunit and kinase domain during nucleotide sensing, and a C-terminal β-binding domain (CTD; residues 396–548). The β subunit (AMPKβ1, 270 aa) contains an N-terminal region (residues 1–67), a carbohydrate-binding module (CBM; residues 68–163), a β-linker that connects the CBM to the C-terminal scaffold, and a C-terminal α/γ-binding domain (residues 184–270), which stabilizes heterotrimer assembly. The γ subunit (AMPKγ1, 331 aa) comprises four cystathionine β-synthase (CBS1–CBS4) repeats organized into two Bateman domains that bind AMP, ADP, and ATP to sense cellular energy status. Together, these structural domains coordinate allosteric regulation, upstream kinase activation, and downstream metabolic signaling.

AMPK is a highly conserved heterotrimeric serine/threonine kinase composed of a catalytic α subunit (AMPKα) and two regulatory subunits, the β (AMPKβ) and γ (AMPKγ) subunits [6]. The coordinated assembly of these three subunits is essential for AMPK activation and function, enabling the kinase to sense changes in cellular energy status and orchestrate metabolic adaptation. The structural organization and functional domains of the AMPK subunits are illustrated in Figure 1.

Mammals express two α isoforms (AMPKα1, 548 amino acids; AMPKα2, 552 amino acids), two β isoforms (AMPKβ1, 270 amino acids; AMPKβ2, 272 amino acids), and three γ isoforms (AMPKγ1, 331 amino acids; AMPKγ2, 569 amino acids; and AMPKγ3, 489 amino acids) [6]. Combinatorial assembly of these isoforms generates up to 12 distinct AMPK heterotrimeric holoenzymes, which exhibit tissue-specific expression patterns and regulatory properties [7]. This isoform diversity enables AMPK to fine-tune metabolic responses according to the physiological demands of different cell types and tissues.

The α subunit contains the N-terminal kinase domain (KD), which catalyzes substrate phosphorylation [6], and a C-terminal regulatory region that mediates heterotrimer assembly. The kinase domain includes the activation loop (A-loop), where phosphorylation of Thr172 by upstream kinases is required for full AMPK activation [8,9]. Located adjacent to the kinase domain is an autoinhibitory domain (AID), which suppresses basal kinase activity, whereas two regulatory subunit-interacting motifs (α-RIM1 and α-RIM2) couple nucleotide sensing by the γ subunit to activation of the catalytic domain [8,9].

The C-terminal domain (CTD) of the AMPK α subunit associates with the C-terminal regions of the β and γ subunits to form the structural core of the heterotrimeric complex, with the β subunit providing the principal scaffold for complex assembly and stability [3,10]. The linker connecting the KD and CTD contains two regulatory subunit-interacting motifs (α-RIM1 and α-RIM2) that couple adenine nucleotide sensing by the γ subunit to activation of the catalytic α subunit [3,11]. Structural studies have shown that these motifs stabilize the active conformation of AMPK by promoting AMP-dependent interactions within the γ subunit and protecting the activating phosphorylation site, Thr172, from dephosphorylation [11]. In addition to nucleotide-dependent regulation, AMPK activity is modulated by mTORC1, which phosphorylates conserved serine residues within the α subunit C-terminal regulatory region (including α1-Ser347 and α2-Ser345), thereby suppressing AMPK activation and providing an additional layer of metabolic regulation [12,13].

The β subunit of AMPK, approximately 270 amino acids in length, serves as the structural scaffold of the heterotrimeric complex, bridging the α and γ subunits to stabilize complex assembly and facilitate inter-subunit communication [11,14]. It contains an N-terminal carbohydrate-binding module (CBM), a flexible linker region, and a C-terminal subunit-interaction domain (SID) that mediates association with the α/γ core [11]. The CBM enables AMPK to bind glycogen particles, thereby linking kinase activity to intracellular glycogen stores and cellular energy availability [15,16]. Glycogen binding through the CBM has been shown to suppress AMPK activation, likely by restricting conformational flexibility or limiting access to upstream kinases, suggesting that glycogen functions as a negative regulator of AMPK signaling [15,16]. Thus, beyond its structural role, the β subunit integrates glycogen sensing with AMPK activation, allowing the kinase to coordinate cellular energy homeostasis in response to nutrient availability.

The AMPK γ subunit functions as the primary energy-sensing component of the AMPK complex, enabling the kinase to respond dynamically to fluctuations in cellular energy status. It contains four cystathionine β-synthase (CBS) motifs organized into two tandem Bateman domains that form the nucleotide-binding core. These CBS motifs bind adenine nucleotides (AMP, ADP, and ATP) to regulate AMPK activity through allosteric mechanisms. Under physiological conditions, CBS4 contains a tightly bound, non-exchangeable AMP molecule, whereas CBS1 and CBS3 serve as exchangeable nucleotide-binding sites that competitively bind AMP, ADP, or ATP. Binding of AMP or ADP at these regulatory sites promotes allosteric activation of AMPK and protects the activating residue Thr172 from dephosphorylation, while ATP antagonizes these effects [17,18].

The γ subunit interacts with the β subunit through a conserved interface within its C-terminal region, thereby contributing to assembly and stability of the heterotrimeric complex [11,19]. Three γ isoforms (γ1, γ2, and γ3) differ in tissue distribution, subcellular localization, and sensitivity to adenine nucleotides, contributing to differences in AMPK regulation across tissues.

### 2.2. AMPK Regulation of Cellular Metabolism

AMPK activation is tightly coupled to the cellular energy status and is triggered by metabolic or environmental stresses that increase the intracellular AMP/ATP and ADP/ATP ratios, including nutrient deprivation, hypoxia, ischemia, excise, or mitochondrial dysfunction [2,20,21]. AMP or ADP binding stabilizes the phosphorylated Thr172 activation loop, thereby prolonging AMPK activity by limiting phosphatase-mediated dephosphorylation [10,18,22].

**Figure 2:**
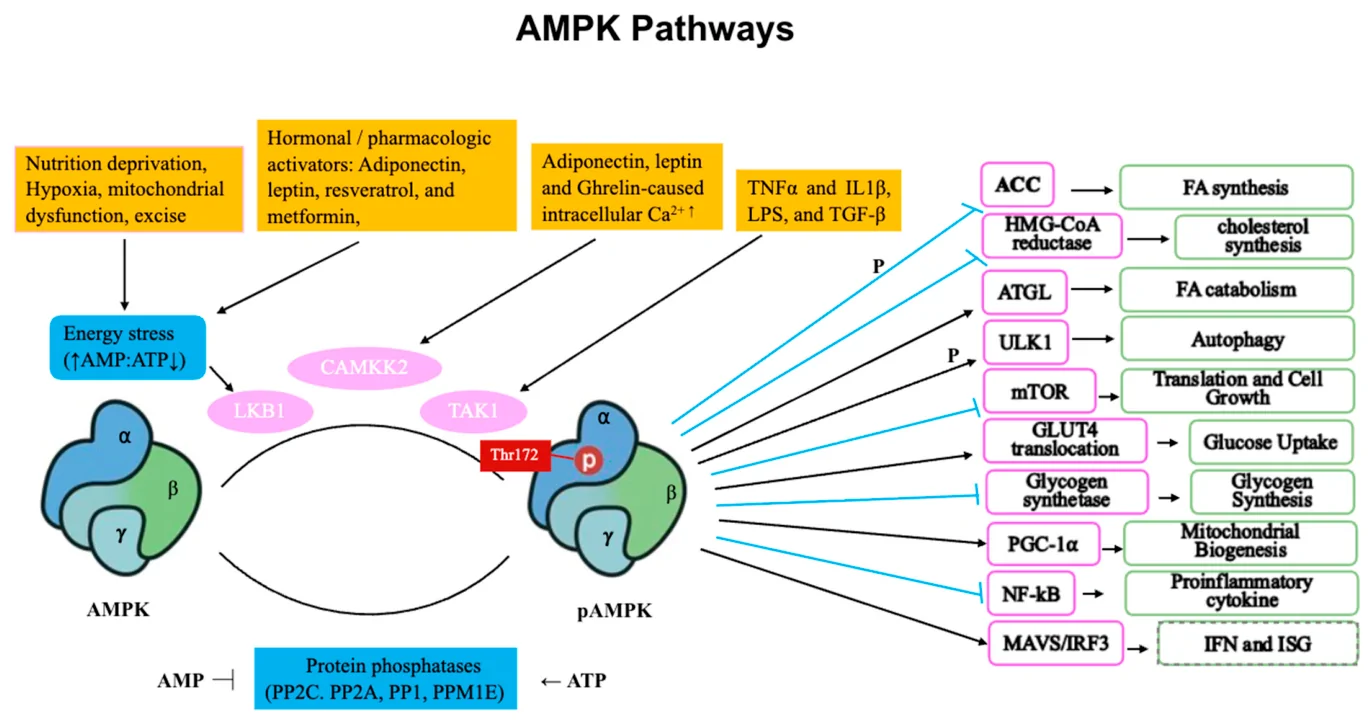
Upstream activation mechanisms and downstream metabolic outputs of AMPK. Energy stress (high AMP:ATP ratio), nutrient deprivation, hypoxia, mitochondrial dysfunction, and exercise activate AMPK through allosteric AMP binding and enhanced LKB1-mediated phosphorylation. Hormonal and pharmacological stimuli, including adiponectin, leptin, metformin, and resveratrol, activate AMPK through signaling pathways involving LKB1 and related upstream regulators, whereas Ca^2+^ mobilization activates AMPK through CaMKK2. In certain cellular contexts, inflammatory stimuli including TNF-α, IL-1β, LPS, and TGF-β can activate AMPK through TAK1-dependent phosphorylation of Thr172. Activated AMPK phosphorylates multiple targets, inhibiting anabolic lipid pathways (ACC; HMG-CoA reductase), suppressing mTOR-dependent growth, and enhancing catabolic processes including FA breakdown (ATGL), autophagy (ULK1), and mitochondrial biogenesis (PGC-1α). AMPK also modulates immunometabolic signaling by suppressing NF-κB and enhancing MAVS/IRF3-mediated interferon responses. Collectively, these outputs link AMPK activity to LD metabolism, β-oxidation, and host antiviral control.

Full activation of AMPK requires phosphorylation at Thr172 by one of several upstream kinases, including liver kinase B1 (LKB1), calcium/calmodulin-dependent protein kinase kinase β (CaMKKβ) [23], and transforming growth factor-β–activated kinase 1 (TAK1) [24] (Figure 2). The specific upstream pathway engaged depends on the nature of the stress stimulus: increases in the AMP/ATP ratio activate LKB1; elevations in intracellular Ca^2+^ levels stimulate CaMKKβ; and inflammatory cytokines to promote AMPK activation through TAK1 [25,26]. Together, these mechanisms enable AMPK to integrate metabolic, calcium, and inflammatory signals, ensuring appropriate cellular adaptation to diverse physiological and pathological stresses.

Once activated, AMPK functions as a central metabolic sensor and master regulator that maintains cellular and organismal energy homeostasis. It restores energy balance by stimulating catabolic pathways that generate ATP—such as glucose uptake, glycolysis, and fatty acid oxidation—while simultaneously suppressing anabolic processes that consume ATP, including lipid, protein, and glycogen synthesis [2,27,28]. Through these actions, AMPK coordinates adaptive responses to energy stress across diverse tissues and cell types, integrating metabolic, nutrient, and stress signaling to promote cell survival and systemic energy balance.

As shown in Figure 2, activated AMPK restores energy homeostasis by promoting catabolic pathways that generate ATP while suppressing anabolic pathways that consume it. It phosphorylates and inactivates acetyl-CoA carboxylase (ACC), thereby reducing fatty acid synthesis and increasing β-oxidation [2]. AMPK also inhibits the mammalian target of rapamycin complex 1 (mTORC1) by phosphorylating TSC2 and Raptor, leading to suppression of protein and lipid biosynthesis [29,30]. In parallel, AMPK enhances glucose uptake (via GLUT4 translocation) and stimulates autophagy through phosphorylation of ULK1 and Beclin-1, linking energy status with organelle quality control [31,32,33]. Through these actions, AMPK orchestrates a global shift toward energy conservation, redox balance, and stress adaptation processes that are commonly hijacked or subverted during viral infections.

## 3. AMPK in Organelle Homeostasis, Stress Response, and Innate Immunity

Beyond its canonical role in energy metabolism, AMPK functions as a central coordinator of organelle homeostasis and cellular stress adaptation. By integrating metabolic, nutrient, and stress signals from mitochondria, the endoplasmic reticulum (ER), lipid droplets (LDs), and lysosomes, AMPK maintains energy balance, redox homeostasis, and organelle quality control across diverse cellular contexts.

AMPK plays a central role in mitochondrial maintenance and quality control by promoting mitochondrial biogenesis through activation of the transcriptional coactivator PGC-1α, which in turn induces downstream transcription factors such as NRF1, NRF2, and TFAM, thereby enhancing oxidative metabolism and cellular ATP production [34,35]. AMPK also regulates mitochondrial dynamics by phosphorylating dynamin-related protein 1 (DRP1), thereby promoting mitochondrial fission in response to energetic stress [36]. Under conditions of mitochondrial damage or energy stress, AMPK activates ULK1 and related autophagy proteins to initiate mitophagy, thereby preserving mitochondrial integrity and function [21,32].

At the ER, AMPK contributes to proteostasis and lipid balance by inhibiting lipid and sterol biosynthesis via phosphorylation of ACC1/2 and suppression of SREBP1 activity, while attenuating ER stress through modulation of metabolic homeostasis [2]. Through these effects, AMPK helps limit excessive biosynthetic stress and supports adaptation to nutrient and energetic imbalance.

LDs represent another key organelle regulated by AMPK. suppressing fatty acid synthesis and lipogenic transcriptional programs, AMPK limits LD biogenesis during energy stress [37,38]. At the same time, AMPK promotes lipid mobilization and fatty acid β-oxidation, enabling stored lipids to be used for ATP production and metabolic adaptation. Through these mechanisms, AMPK coordinates functional crosstalk among LDs, mitochondria, and the ER—three organelle hubs that are central to lipid metabolism, redox regulation, and antiviral signaling.

AMPK also modulates innate immunity. AMPK activation can support antiviral defense by maintaining mitochondrial fitness and metabolic conditions favorable for interferon signaling. In parallel, AMPK restrains excessive inflammation by inhibiting NF-κB activation and downstream proinflammatory cytokine expression [39]. These dual activities allow AMPK to promote antiviral responses while limiting immunopathology.

Collectively, AMPK acts as a master regulator linking metabolic sensing, organelle communication, and immune signaling. Many RNA viruses, including flaviviruses such as Zika virus (ZIKV) and dengue virus (DENV), have evolved strategies to manipulate the AMPK–mTOR and AMPK–LD signaling pathways to remodel organelle architecture, redirect lipid metabolism, suppress antiviral responses, and create a cellular environment favorable for viral replication.

## 4. AMPK-Mediated Antiviral Mechanisms

Although flaviviruses frequently suppress AMPK signaling to establish a metabolically favorable environment for replication, activation of AMPK can restrict viral infection through multiple complementary mechanisms. Beyond its role in energy homeostasis, AMPK coordinates cellular antiviral defenses by regulating autophagy, mitochondrial quality control, redox homeostasis, lipid metabolism, and innate immune signaling. Together, these pathways preserve cellular homeostasis while limiting the metabolic resources and organelle remodeling required for efficient flavivirus replication.

### 4.1. AMPK Activation and Autophagy

Autophagy is an evolutionarily conserved catabolic pathway that degrades and recycles cytoplasmic components, thereby maintaining cellular energy balance, organelle quality, and protein homeostasis. As a master metabolic sensor, AMPK is a principal inducer of autophagy. Upon activation, AMPK directly phosphorylates the autophagy-initiating kinase ULK1, which subsequently activates Beclin-1 and the VPS34 class III phosphatidylinositol 3-kinase complex to initiate phagophore formation and autophagosome biogenesis [40,41]. AMPK also promotes autophagy indirectly by inhibiting mTORC1, thereby relieving its inhibitory effect on the ULK1 complex. Through these coordinated mechanisms, the AMPK–mTOR–ULK1 signaling axis links cellular energy status to autophagic flux.

The role of autophagy during flavivirus infection is highly context-dependent. During the early stages of infection, flaviviruses such as DENV and ZIKV exploit components of the autophagic machinery to mobilize lipid droplets through lipophagy, generating free fatty acids that fuel mitochondrial β-oxidation and support viral replication. In contrast, sustained activation of AMPK can shift autophagy toward an antiviral program by enhancing autophagic flux, preserving organelle homeostasis, and limiting the lipid and energy resources required for viral replication. AMPK-dependent autophagy may also facilitate the clearance of damaged organelles and viral components, thereby reducing cellular stress and reinforcing antiviral defense. Consistent with this model, pharmacological activation of AMPK using AICAR, metformin, GSK621, or PF-06409577 suppresses DENV and ZIKV replication in multiple experimental systems [42,43].

Although the magnitude of these antiviral effects varies with cell type, metabolic state, and stage of infection, the overall evidence supports AMPK activation as a host-directed strategy that counteracts flavivirus-induced metabolic reprogramming. By simultaneously suppressing lipogenesis, promoting autophagy, and restoring metabolic homeostasis, the AMPK–mTOR–ULK1 pathway represents an attractive therapeutic target for broad-spectrum antiviral intervention.

### 4.2. AMPK Regulation of Mitochondrial Function and Ros Homeostasis

Flavivirus infection is frequently associated with mitochondrial dysfunction, excessive reactive oxygen species (ROS) production, and metabolic stress. AMPK is a central regulator of mitochondrial homeostasis, coordinating mitochondrial biogenesis, quality control, and oxidative metabolism in response to energetic stress. Upon activation, AMPK promotes mitochondrial biogenesis by stimulating the transcriptional coactivator PGC-1α, which enhances oxidative phosphorylation and ATP production. AMPK also preserves mitochondrial quality by activating mitophagy through phosphorylation of ULK1 and mitofusin-2 (MFN2), facilitating the selective removal of damaged mitochondria and maintenance of mitochondrial network integrity [2,32,44].

Mitochondrial dysfunction is a common feature of flavivirus infection. During ZIKV infection, mitochondrial fragmentation, loss of mitochondrial membrane potential, and elevated ROS production are associated with impaired AMPK signaling, contributing to cellular stress and viral pathogenesis [42]. Pharmacological activation of AMPK with metformin, AICAR, or GSK621 restores mitochondrial function, reduces oxidative stress, and suppresses ZIKV replication in multiple cell models [42]. Similarly, in Japanese encephalitis virus (JEV) infection, activation of AMPK-dependent mitochondrial protective pathways preserves mitochondrial integrity, limits neuronal apoptosis, and mitigates virus-induced neuropathology [2].

Collectively, these findings indicate that AMPK functions as a key regulator of mitochondrial quality control during flavivirus infection. By maintaining mitochondrial bioenergetics, promoting mitophagy, and limiting ROS accumulation, AMPK activation creates an intracellular environment that is less permissive for viral replication while protecting host cells from virus-induced metabolic and mitochondrial damage.

### 4.3. Crosstalk Between AMPK and Innate Immune Signaling

Beyond its metabolic functions, AMPK has emerged as an important regulator of innate antiviral immunity by coordinating mitochondrial function, cellular metabolism, and inflammatory signaling. AMPK enhances antiviral defense by promoting mitochondrial antiviral-signaling protein (MAVS)-dependent activation of the RIG-I-like receptor (RLR)–TBK1–IRF3 pathway, thereby stimulating the production of type I interferons (IFN-I) and interferon-stimulated genes (ISGs) [45]. Maintenance of mitochondrial integrity by AMPK further supports MAVS signaling, highlighting the close interplay between mitochondrial quality control and antiviral immunity.

In addition to promoting antiviral responses, AMPK limits excessive inflammation by suppressing NF-κB activation and inhibiting NLRP3 inflammasome assembly, thereby reducing the production of proinflammatory cytokines and preventing immunopathology [39,46]. Through these complementary actions, AMPK coordinates an effective antiviral response while maintaining inflammatory homeostasis.

Although the immunomodulatory functions of AMPK during flavivirus infection are not yet fully defined, accumulating evidence indicates that flaviviruses manipulate AMPK signaling to dampen innate immune responses while simultaneously reprogramming host metabolism to support replication. This dual regulation of antiviral interferon signaling and inflammatory homeostasis may contribute to restricting flavivirus replication and limiting virus-induced tissue damage [45]. Conversely, pharmacological activation of AMPK enhances interferon signaling, promotes ISG expression, preserves mitochondrial function, and suppresses excessive inflammatory responses, collectively creating a cellular environment that is less permissive for viral replication. Thus, AMPK serves as a critical molecular link between metabolic adaptation and innate immunity, making it an attractive target for host-directed antiviral therapy against flavivirus infections.

## 5. Regulation of AMPK by Flaviviruses

**Figure 3:**
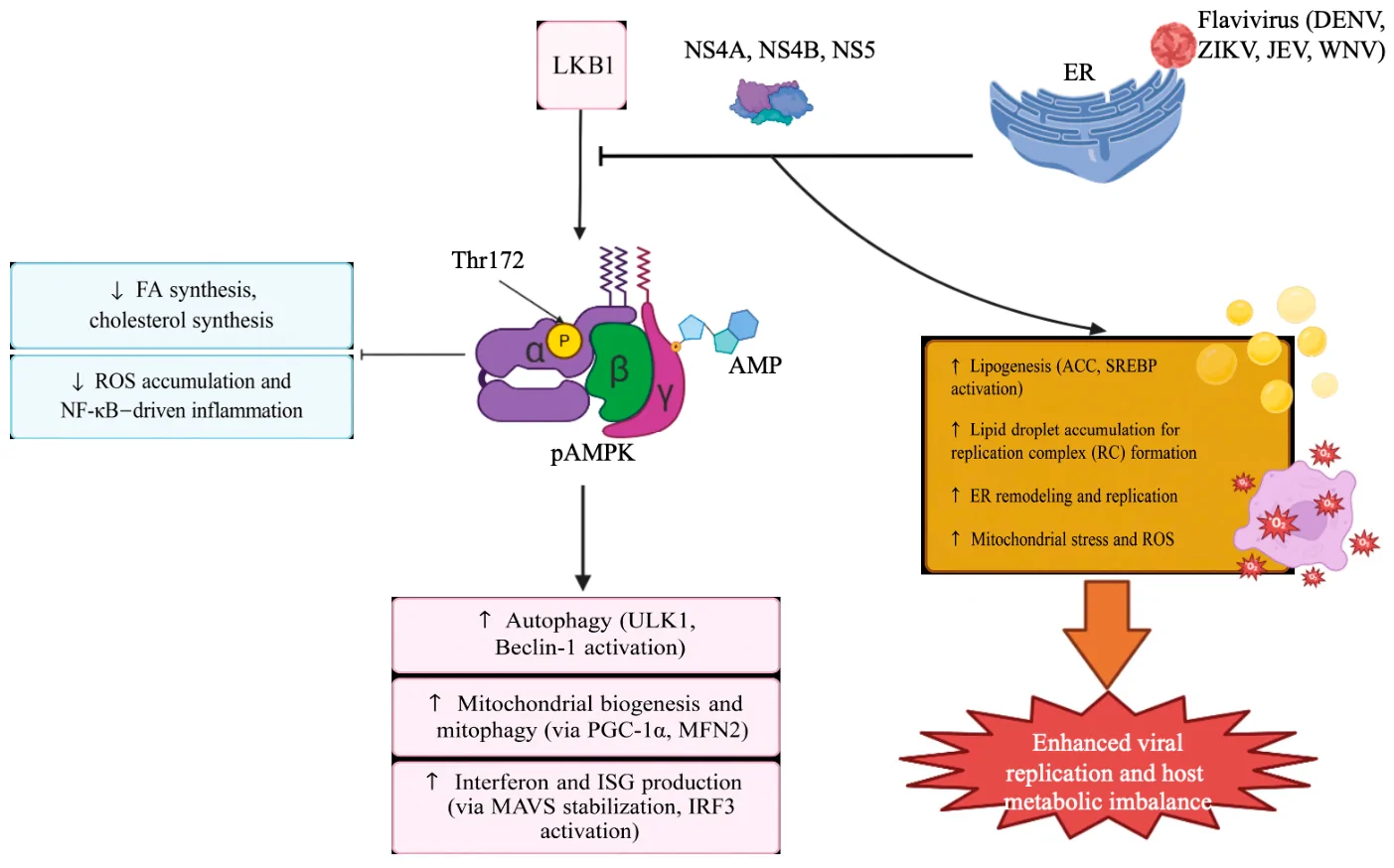
AMPK-driven metabolic control and flavivirus-mediated rewiring of host pathways. Schematic illustration of the reciprocal interactions between AMPK signaling and flavivirus infection. AMPK regulates lipid metabolism, autophagy, mitochondrial homeostasis, and innate immune responses, whereas flaviviruses, including DENV, ZIKV, JEV, and WNV, manipulate these pathways through viral proteins such as NS4A, NS4B, NS5, and capsid to promote replication and evade host defenses.

Flavivirus replication imposes substantial energetic and biosynthetic demands on host cells, driving metabolic reprogramming that frequently involves modulation of AMPK signaling. As summarized in Figure 3, AMPK functions as a central metabolic checkpoint during infection by promoting catabolic pathways, mitochondrial maintenance, autophagy, and antiviral interferon signaling while suppressing lipid biosynthesis and excessive inflammatory responses. However, the impact of flavivirus infection on AMPK activity is highly context-dependent and varies according to the viral species, strain, cell type, metabolic state, and stage of infection.

### 5.1. AMPK Modulation During Dengue Virus (DENV) Infection

The regulation of AMPK during dengue virus (DENV) infection remains complex and somewhat controversial. This complexity has been comprehensively discussed in previous reviews [47]. AMPK controls cellular energy homeostasis and lipid metabolism by regulating lipogenesis, fatty acid β-oxidation, autophagy, and LD dynamics, all of which influence DENV replication and assembly.

Jordan and colleagues reported that DENV-2 infection activates AMPK in HepG2 cells, coinciding with inhibition of mTORC1 signaling and induction of proviral lipophagy [48]. Although mTORC1 suppression was found to occur independently of AMPK, these findings suggest that DENV can coordinate multiple metabolic checkpoints to mobilize lipid stores. Lipophagy-derived free fatty acids may fuel mitochondrial β-oxidation and ATP production, thereby supporting viral replication under energy-demanding conditions.

In contrast, Soto-Acosta et al. demonstrated that DENV-2 impairs AMPK activation in Huh7 cells, leading to increased activity of 3-hydroxy-3-methylglutaryl-CoA reductase (HMGCR) and enhanced cholesterol biosynthesis [49]. Elevated cholesterol can support DENV replication by enriching ER-derived membranes and LD-associated platforms required for replication complex formation. These apparently opposing observations—AMPK activation versus AMPK suppression—likely reflect differences in viral strain, infection timing, experimental conditions, or host-cell metabolic state.

Pharmacological studies further support an antiviral role for AMPK activation during DENV infection. The selective AMPK activator PF-06409577 suppresses DENV replication [50], and the antidiabetic drug metformin, an indirect AMPK activator, reduces DENV replication in hepatocytes and monocytes [42,51]. Mechanistically, AMPK activation may restrict DENV by suppressing lipid and cholesterol biosynthesis, promoting lipid catabolism, altering LD dynamics, and limiting the lipid resources required for viral replication, assembly, and maturation.

Together, these studies indicate that AMPK has a context-dependent role during DENV infection. While DENV may transiently exploit AMPK-linked catabolic pathways such as lipophagy to generate energy substrates, sustained AMPK activation generally appears to impose antiviral pressure by limiting lipid availability and reinforcing metabolic stress responses. Further work is needed to define the temporal regulation of AMPK signaling during DENV infection and to determine how viral strain, cell type, and host metabolic status shape AMPK-dependent outcomes.

### 5.2. Zika Virus (Zikv) Interference with AMPK and Lipid Metabolism

Zika virus (ZIKV) is a neurotropic and cardiotropic flavivirus associated with congenital abnormalities, neurological disease, and cardiovascular complications. Like other flaviviruses, ZIKV profoundly remodels host metabolism to support viral replication. ZIKV relies on lipid droplets (LDs) and endoplasmic reticulum (ER)-derived membranes for replication complex formation, viral RNA synthesis, and progeny virion assembly. Disruption of AMPK signaling is a key component of this metabolic rewiring.

ZIKV infection suppresses AMPK activation, leading to reduced phosphorylation and increased activity of acetyl-CoA carboxylase (ACC). Because ACC promotes fatty acid synthesis, its activation enhances de novo lipogenesis and LD accumulation, creating a lipid-enriched environment that supports replication complex biogenesis and viral replication. Mechanistically, ZIKV nonstructural proteins NS4A and NS4B have been shown to impair AMPK signaling and mitochondrial integrity, resulting in metabolic stress, reduced ATP production, mitochondrial dysfunction, and apoptosis [50,52].

Pharmacological and genetic studies support an antiviral role for AMPK during ZIKV infection. The AMPK activator PF-06409577, which also inhibits DENV replication, suppresses ZIKV replication [50]. Consistently, ZIKV infection induces a time-dependent decrease in phosphorylated AMPK and its downstream substrate phospho-ACC, indicating active viral suppression of AMPK signaling [53]. Activation of AMPK by AICAR, metformin, or the specific AMPKα activator GSK621 markedly reduces ZIKV replication, whereas the AMPK inhibitor compound C restores viral growth [53]. In agreement, AMPK knockdown by lentiviral shRNA or loss of AMPKα in mouse embryonic fibroblasts enhances ZIKV replication, further supporting AMPK as an antiviral metabolic restriction factor.

The antiviral effects of AMPK activation extend beyond lipid metabolism. AMPK stimulation enhances the expression of interferon (IFN) and IFN-stimulated gene (ISGs) expression, including OAS2, ISG15, and MX1, while suppressing proinflammatory cytokines such as TNF-α and CCL5 [53]. Thus, AMPK links metabolic restriction with innate antiviral defense by limiting lipid availability, preserving mitochondrial function, promoting autophagy, and reinforcing IFN-mediated antiviral signaling.

Additional studies support the protective role of AMPK-associated pathways in ZIKV infection. Folic acid, which promotes AMPK activation and mitochondrial function, was reported to reduce vertical ZIKV transmission from infected dams to fetuses [54]. More recently, DISC1 (Disrupted-in-Schizophrenia 1) was shown to exert anti-ZIKV activity by enhancing AMPKα phosphorylation, promoting autophagy, and suppressing mTOR signaling [55].

Collectively, these findings indicate that ZIKV suppresses AMPK signaling to promote lipogenesis, LD remodeling, mitochondrial dysfunction, and viral replication. Conversely, AMPK activation restores metabolic balance, enhances autophagy and antiviral gene expression, and limits ZIKV replication. Targeting AMPK-regulated lipid metabolism and organelle homeostasis may therefore represent a promising host-directed strategy for controlling ZIKV infection and reducing virus-associated neurological and cardiovascular pathology.

### 5.3. WNV, JEV, and Other Flaviviruses

Like DENV and ZIKV, other flaviviruses, including West Nile virus (WNV), Japanese encephalitis virus (JEV), and yellow fever virus (YFV), remodel host metabolism to support replication. These viruses rely on lipid biosynthesis, endoplasmic reticulum (ER) membrane expansion, and lipid droplet (LD) remodeling to generate replication complexes. In many contexts, this metabolic reprogramming is associated with suppression of AMPK signaling, which favors a lipid-rich and energetically permissive environment for viral replication.

For WNV, the capsid (C) protein promotes ubiquitination and proteasomal degradation of AMPK, thereby suppressing AMPK signaling [56]. Loss of AMPK activity impairs autophagy, leading to accumulation of misfolded protein aggregates, increased cellular stress, and disrupted organelle quality control. These effects may contribute to neuronal injury and neuropathogenesis during WNV-associated encephalitis.

JEV infection disrupts mitochondrial homeostasis in neuronal cells, resulting in mitochondrial dysfunction, oxidative stress, altered mitochondrial dynamics, loss of mitochondrial membrane potential, and apoptosis [57]. These pathogenic effects have been associated with dysregulated calcium signaling and neuroinflammatory pathways. Because AMPK is a central regulator of mitochondrial homeostasis and biogenesis, pharmacological activation of AMPK has been proposed as a potential strategy to restore mitochondrial function and suppress JEV replication [58].

For other flaviviruses, including YFV, available evidence suggests that viral replication is often associated with enhanced lipogenesis and reduced AMPK activity, although the underlying mechanisms remain incompletely defined. Conversely, AMPK activation has demonstrated antiviral and cytoprotective effects in several flavivirus infection models [59]. For example, ginkgolic acid, a natural compound derived from *Ginkgo biloba*, inhibits ZIKV entry and membrane fusion. Although the antiviral study did not directly investigate AMPK signaling, previous work has shown that ginkgolic acid activates AMPK and suppresses lipogenesis, suggesting a potential mechanism that warrants further investigation [59].

### 5.4. Hepatitis C Virus (HCV) and AMPK

Although hepatitis C virus (HCV) is classified within the family *Flaviviridae*, it belongs to the genus *Hepacivirus* and differs biologically from mosquito-borne flaviviruses such as DENV and ZIKV [60,61]. HCV exhibits strong hepatotropism and is highly dependent on liver-specific lipid metabolism and host factors. Chronic HCV infection is closely associated with metabolic dysregulation, including hepatic steatosis, insulin resistance, and altered lipid and glucose homeostasis, which contribute to chronic hepatitis, cirrhosis, and hepatocellular carcinoma [62].

HCV infection inhibits AMPK activation, promoting a lipogenic environment favorable for viral replication [63]. Mechanistically, HCV induces phosphorylation of AMPK at serine 485 by protein kinase B (PKB/AKT), which interferes with phosphorylation at the activation site threonine 172, resulting in AMPK inactivation [62]. This metabolic shift enhances fatty acid synthesis and lipid droplet accumulation, providing lipid-rich platforms that support HCV replication, assembly, and release.

Additional studies indicate that the relationship among HCV, AMPK, AKT/PKB, and autophagy is context-dependent. In Huh7 cells, HCV infection has been reported to inhibit both AMPK and PKB while promoting autophagy, a process that can support viral replication by mobilizing lipid droplets and contributing to double-membrane vesicle formation [64]. These findings indicate that HCV dynamically modulates AMPK signaling depending on the infection stage, cellular metabolic status, and experimental context.

At the viral protein level, NS5A has been implicated in AMPK suppression and lipogenic activation. NS5A inhibits AMPK and upregulates SREBP-1c, ACC1, and fatty acid synthase (FASN), thereby promoting triglyceride and free fatty acid accumulation, lipid droplet expansion, and HCV-associated steatosis [65]. In addition, HCV proteins such as NS3 and NS5A modulate PKCδ–HuR signaling to promote cytoplasmic translocation of the RNA-binding protein HuR, facilitating viral RNA metabolism, assembly, and release. Although this pathway is not a direct component of canonical AMPK signaling, it cooperates with HCV-induced metabolic reprogramming by supporting a cellular environment favorable for viral replication.

Pharmacological restoration of AMPK activity restricts HCV replication. Metformin, a widely used antidiabetic drug and indirect AMPK activator, restores AMPK phosphorylation, suppresses de novo lipogenesis, enhances fatty acid oxidation, and reduces HCV replication [66]. Similarly, other AMPK-activating strategies can disrupt the lipid droplet environment required for efficient HCV assembly.

Collectively, these findings establish AMPK as a metabolic checkpoint that counteracts HCV-driven lipogenesis, LD biogenesis, and autophagy. By suppressing AMPK, HCV hijacks hepatic lipid metabolism to support its replication cycle. Conversely, AMPK activation through pharmacological agents such as metformin or AICAR not only restricts viral replication but also mitigates virus-induced metabolic and inflammatory pathology, highlighting its potential as a dual-acting antiviral and metabolic therapeutic target in chronic HCV infection.

Collectively, these studies highlight AMPK as a conserved host defense factor that maintains energy homeostasis, supports mitochondrial quality control, promotes autophagy, and limits viral replication. Flaviviruses counteract this restriction through diverse mechanisms, including AMPK degradation, dephosphorylation, or suppression of downstream signaling. Thus, restoring AMPK activity may not only inhibit viral replication but also protect against virus-induced metabolic dysfunction and neurodegenerative damage.

## 6. Therapeutic Targeting of AMPK in Flavivirus Infection

Given its central role in coordinating metabolism, organelle homeostasis, and innate immunity, AMPK has emerged as a promising host-directed antiviral target. Pharmacological activation of AMPK may restrict flavivirus replication by suppressing anabolic lipid metabolism, promoting fatty acid oxidation and autophagy, preserving mitochondrial function, and enhancing antiviral immune responses. Because several AMPK-modulating compounds are already clinically approved or under investigation for metabolic diseases, this pathway offers opportunities for therapeutic repurposing against flavivirus infections.

### 6.1. Pharmacological Activators of AMPK

Pharmacological activation of AMPK represents a practical approach to harness host metabolic control for antiviral intervention. AMPK activators act through distinct mechanisms, including direct allosteric activation, modulation of upstream kinases, or indirect induction of energetic stress. Despite these mechanistic differences, they converge on a common metabolic outcome: suppression of ATP-consuming anabolic pathways and enhancement of catabolic processes that are less permissive to viral replication. In flavivirus infection, AMPK activation can inhibit lipid and cholesterol biosynthesis, enhance fatty acid β-oxidation, promote autophagy, preserve mitochondrial function, and modulate innate immune signaling. Representative AMPK activators, their mechanisms of action, molecular targets, and antiviral relevance are summarized in Table 1.

Among available AMPK activators, AICAR and metformin currently provide the strongest experimental support for antiviral activity in flavivirus systems. AICAR is converted intracellularly to the AMP mimetic ZMP, which binds the AMPK γ subunit and promotes AMPK activation, thereby mimicking cellular energy stress [75]. By shifting metabolism away from biosynthesis and toward energy conservation, AICAR can restrict the metabolic capacity required for viral genome replication and virion production.

Metformin, a clinically approved antidiabetic drug, activates AMPK indirectly by inhibiting mitochondrial complex I, increasing the AMP/ATP ratio and stimulating AMPK-dependent metabolic adaptation [76]. In flavivirus infection models, metformin reduces dengue virus replication and has been associated with decreased virus-induced cellular stress and inflammatory responses. Its established clinical use, safety profile, and immunometabolic effects make metformin an attractive candidate for repurposing, although efficacy will likely depend on timing, tissue distribution, and host metabolic status.

Additional AMPK-activating compounds also show antiviral potential, although their mechanisms in flavivirus systems require further clarification. Resveratrol activates AMPK indirectly through the SIRT1–LKB1 axis and may inhibit flavivirus replication by improving mitochondrial function, enhancing oxidative metabolism, and limiting inflammatory responses [77]. Salicylate, the active metabolite of aspirin, directly activates AMPK via the β subunit and exhibits broad antiviral activity against RNA viruses, supporting the concept that AMPK-dependent metabolic restriction can limit viral replication [2]. Berberine activates AMPK through mitochondrial perturbation and AMP accumulation and has been reported to RNA viruses disrupting lipid biosynthesis and cellular energetic pathways required for replication [39,78].

Collectively, these studies support AMPK activation as a promising host-directed antiviral strategy. AICAR and metformin remain the most experimentally supported candidates, whereas resveratrol, salicylate, berberine, and newer direct AMPK activators warrant further investigation. Future studies should define the optimal timing, dose, tissue specificity, and combination strategies needed to maximize antiviral efficacy while minimizing unwanted metabolic effects.

### 6.2. Context-Dependent Effects of AMPK Modulation During Flavivirus Infection

Although AMPK activation generally restricts flavivirus replication, its effects are highly context-dependent and influenced by the stage of infection, cell type, tissue metabolic state, and the specific virus involved [43,79]. During the early phase of infection, several flaviviruses transiently exploit AMPK-regulated catabolic pathways, particularly autophagy and lipophagy, to mobilize lipid stores and generate ATP and biosynthetic intermediates required for viral RNA replication and replication organelle biogenesis. Under these conditions, moderate or transient AMPK activation may inadvertently support viral replication by enhancing metabolic adaptation and substrate availability [80,81].

As infection progresses, however, sustained AMPK activation shifts cellular metabolism toward an antiviral state by suppressing de novo lipogenesis, promoting fatty acid β-oxidation, preserving mitochondrial function, and enhancing innate immune signaling [2,78]. This metabolic reprogramming limits the lipid and energy resources required for viral replication while promoting autophagy, mitochondrial quality control, and interferon-mediated antiviral responses. Consequently, the biological outcome of AMPK activation reflects a dynamic balance between virus-driven metabolic exploitation and host-directed metabolic restriction.

These observations underscore the importance of considering the timing, duration, and magnitude of AMPK activation when developing therapeutic strategies. Effective antiviral interventions will likely require precise modulation of AMPK activity to maximize antiviral efficacy while minimizing the possibility of inadvertently supporting early viral replication.

### 6.3. AMPK as a Host-Targeted Antiviral Strategy: Opportunities and Challenges

Targeting AMPK represents an attractive host-directed antiviral strategy because it simultaneously regulates multiple cellular pathways required for flavivirus replication, including lipid metabolism, autophagy, mitochondrial homeostasis, and innate immune signaling. Unlike direct-acting antivirals that target viral proteins, AMPK-directed therapies are less susceptible to the emergence of antiviral resistance and may provide broad-spectrum activity against multiple flaviviruses that share common metabolic dependencies. In addition to suppressing viral replication, restoration of AMPK activity may alleviate virus-induced metabolic dysfunction, oxidative stress, and excessive inflammatory responses, thereby reducing disease severity.

Despite these advantages, several challenges remain. Because AMPK regulates numerous physiological processes, prolonged or systemic activation could disrupt normal anabolic metabolism, alter glucose and lipid homeostasis, or interfere with tissue-specific metabolic functions. Furthermore, the effects of AMPK modulation vary among viruses, cell types, and stages of infection, indicating that therapeutic efficacy will depend on careful optimization of treatment timing, dosage, and tissue distribution.

Future efforts should focus on the development of selective and tissue-targeted AMPK modulators, together with combination strategies that integrate AMPK activators with direct-acting antivirals, immunomodulators, or other host-directed therapies. A deeper understanding of the temporal regulation of AMPK signaling, its crosstalk with organelle homeostasis and innate immunity, and its virus-specific functions will be critical for translating AMPK-targeted therapies into effective broad-spectrum antiviral interventions against flavivirus infections.

## 7. Concluding Remarks and Future Directions

AMPK has emerged as a central regulator of the metabolic and signaling networks that govern the outcome of flavivirus infection. Beyond its classical role as a cellular energy sensor, AMPK coordinates lipid metabolism, autophagy, mitochondrial homeostasis, endoplasmic reticulum (ER) function, and innate immune signaling, thereby integrating cellular metabolism with antiviral defense. Throughout their replication cycle, flaviviruses extensively reprogram these AMPK-regulated pathways to remodel intracellular membranes, expand lipid droplets, optimize energy production, and evade host immune responses. Consequently, the balance between virus-driven metabolic rewiring and AMPK-mediated metabolic restriction is a key determinant of viral replication, cellular adaptation, and disease pathogenesis.

Accumulating evidence indicates that pharmacological activation of AMPK suppresses flavivirus replication by simultaneously inhibiting lipogenesis, promoting fatty acid β-oxidation, restoring mitochondrial function, enhancing autophagy, and strengthening interferon-mediated antiviral responses. These multifaceted activities distinguish AMPK from conventional antiviral targets and position it as an attractive candidate for host-directed antiviral therapy with the potential for broad-spectrum activity against multiple flaviviruses.

Despite these advances, several important questions remain unresolved. First, the temporal dynamics of AMPK signaling during flavivirus infection remain poorly understood, particularly the transition from early virus-induced metabolic adaptation to later antiviral restriction. Second, the molecular mechanisms by which individual viral proteins manipulate AMPK and its downstream metabolic networks require further investigation. Third, the interactions among AMPK, lipid droplets, ER remodeling, mitochondrial quality control, and innate immune signaling have yet to be integrated into a unified model of flavivirus-induced metabolic reprogramming. Finally, future preclinical studies should evaluate selective and tissue-specific AMPK modulators, either alone or in combination with direct-acting antivirals and immunomodulators, using physiologically relevant animal models and human organoid systems.

In summary, AMPK functions as a master metabolic hub linking energy sensing, organelle homeostasis, and innate immunity during flavivirus infection. A deeper understanding of how flaviviruses exploit this regulatory network will not only provide new insights into virus–host interactions but also accelerate the development of innovative host-directed therapies with broad-spectrum antiviral potential.

## Figures and Tables

**Table 1:** Pharmacological activation of AMPK in flavivirus infection. Representative AMPK-activating compounds, their mechanisms of activation, molecular targets, and reported antiviral effects. These agents illustrate how modulation of host metabolism may be leveraged as a host-directed therapeutic strategy against flaviviruses. AICAR: 5-aminoimidazole-4-carboxamide ribonucleotide. AICAR.

Compound	Activation Mechanism	Molecular Target	Antiviral Evidence	Functional Outcome in Flavivirus Context	References
AICAR	AMP mimetic; intracellular conversion to ZMP activates AMPK	AMPK γ subunit (AMP-binding site)	Suppresses DENV and ZIKV replication	Restores cellular energy balance and inhibits lipogenesis, limiting lipid-dependent viral replication	[53,67,68,69]
Metformin	Indirect activation via inhibition of mitochondrial complex I, increasing AMP:ATP ratio	Mitochondrial respiratory chain (Complex I)	Reduces DENV replication in hepatocytes; attenuates ZIKV-induced neuronal damage	Induces energetic stress and restricts biosynthetic pathways required for viral replication; confers cytoprotective effects	[42,70]
Resveratrol	Indirect activation via SIRT1–LKB1 axis	SIRT1-mediated AMPK activation	Inhibits ZIKV and DENV replication and associated neuroinflammation	Enhances AMPK–PGC-1α signaling, promoting mitochondrial function and suppressing inflammatory and metabolic conditions favorable for viral replication	[71,72,73]
Salicylate	Direct allosteric activation	AMPK β subunit	Broad antiviral effects in RNA viruses	Activates AMPK-dependent metabolic restriction and modulates host inflammatory responses	
Berberine	Indirect activation via mitochondrial perturbation and AMP accumulation	Mitochondria-associated pathways	Inhibits replication of multiple RNA viruses	Disrupts lipid biosynthesis and cellular energetics required for viral replication	[74]

## Data Availability

No new data were created or analyzed in this study. Data sharing is not applicable to this article.
